# Supplementary material for: Nonlinear relationships of fibrin network structure as a function of fibrinogen and thrombin concentrations for purified fibrinogen and plasma clots
Source: Acta Biomater. Author manuscript; Available in PMC 2026 Nov 10. (PMC13749047; doi:10.1016/j.actbio.2026.06.047)
Supplement: Supplementary file 1 [file NIHMS2215807-supplement-Cai_et_al_Complete_Supplement.pdf]

Supplement

Nonlinear Relationships of Fibrin Network Structure as a Function of Fibrinogen and Thrombin Concentrations for Purified Fibrinogen and Plasma Clots

Can Cai[1]*, Zezhong Zhang[1]*, Arezoo Nameny[1], Daniel Nelli[1], Keith Bonin[1], Brittany E. Bannish[2], Nathan E. Hudson[3], Glen Marrs[4], Stephen R. Baker[1†], Martin Guthold[1†]

[1] Department of Physics, Wake Forest University, Winston-Salem, NC 27109

[2] Department of Mathematics and Statistics, University of Central Oklahoma, Edmond, Oklahoma, 73034

[3] Department of Physics, East Carolina University, Greenville, NC 27885

[4] Department of Biology, Wake Forest University, Winston-Salem, NC 27109

* These authors contributed equally to this work.

Table of Contents

S1. Power Law Justification

S2. Structural Median or Mean Data for each Fibrinogen and Thrombin Concentration

S3. Additional Analysis to Test Robustness of Statistics (Leave-One-Condition-Out Analysis)

S4. Turbidity Analysis of Fibrin Networks Formation with Different Amounts of Added Alexa-488-Labeled Fibrinogen

S1. Power Law Justification

S1.1. Michaelis–Menten fibrinogen to fibrin conversion with fibrin depletion

We model thrombin cleavage of fibrinogen and subsequent incorporation of fibrin species into growing structures. Let

$F(t) \equiv [\mathrm{Fgn}](t)$: fibrinogen concentration (substrate)

$A(t)$: effective pool of incorporation-competent fibrin species produced by cleavage (fibrin monomers/small oligomers available for incorporation)

$N(t)$: density of reactive growth interfaces (effective growth units/branch sites; a proxy for total reactive interfaces available for incorporation)

$[\mathrm{Thr}]_0$: initial thrombin concentration

$[\mathrm{Thr}]_{\mathrm{eff}}(t) = [\mathrm{Thr}]_0\, a(t)$: effective thrombin level, with $0 < a(t) \leq 1$ capturing inhibition/binding/sequestration without implying stoichiometric thrombin consumption [1]

k_{cat} and K_m: Michaelis–Menten parameters for thrombin cleavage of fibrinogen

k_{el}: effective incorporation (elongation) rate constant

k_{nuc}: effective rate constant for creation of new growth interfaces

n: effective exponent summarizing the dominant pathway(s) for interface creation (a coarse-grained, not necessarily integer "order")

t_*: fixed readout time

t_w: onset time of the main structure-building window (defined phenomenologically below)

A minimal Michaelis–Menten description of the production rate is

$$v_\mathrm{prod}(t) = \frac{k_\mathrm{cat}\,[\mathrm{Thr}]_\mathrm{eff}(t)\,F(t)}{K_m + F(t)}. \qquad \text{(S1)}$$

Substrate depletion is retained explicitly:

$$\frac{dF}{dt} = -v_\mathrm{prod}(t). \qquad \text{(S2)}$$

S1.2. Mass balance for $A(t)$ and mass-action incorporation

Fibrin species are produced and incorporated into growing structures:

$$\frac{dA}{dt} = v_\mathrm{prod}(t) - v_\mathrm{cons}(t). \qquad \text{(S3)}$$

We use a minimal mass-action [2] form for incorporation:

$$v_\mathrm{cons}(t) = k_\mathrm{el}\,A(t)\,N(t). \qquad \text{(S4)}$$

S1.3. Effective creation of growth interfaces

We coarse-grain the creation of new reactive interfaces as

$$\frac{dN}{dt} = k_{\text{nuc}}\, A(t)^{n}, \qquad \text{(S5)}$$

where n summarizes the dominant pathway(s) generating interfaces in an effective description [3].

S1.4. Main structure-building window and quasi-steady clamping of $\boldsymbol{A(t)}$

The early-time kinetics of fibrin generation and incorporation are complex and not fully resolved. We therefore introduce a phenomenological main structure-building window $[t_w,\ t_*]$, defined as the interval that contributes most strongly to the structural readouts at t_*. Quasi-steady-state (QSSA) reductions are widely used in enzyme/biochemical kinetics to eliminate fast-relaxing intermediate species [4]; here we use this idea as an effective approximation to remove $A(t)$ from the dynamics and obtain analytic power-law scalings. Specifically, during $[t_w,\ t_*]$ we assume production and incorporation are of comparable magnitude, so the activated pool $A(t)$ is approximately clamped and satisfies a production–consumption balance:

$$\frac{dA}{dt} \approx 0 \Rightarrow A(t) \approx \frac{v_{\text{prod}}(t)}{k_{\text{el}}\, N(t)}, t \in [t_{\text{w}}, t_*]. \qquad \text{(S6)}$$

Substituting Eq. (S6) into Eq. (S5) gives, for $t \in [t_{\text{w}}, t_*]$,

$$\frac{dN}{dt} = k_{\text{nuc}} \left(\frac{v_{\text{prod}}(t)}{k_{\text{el}}\, N(t)}\right)^{n} \Rightarrow N(t)^{n}\, \frac{dN}{dt} = \frac{k_{\text{nuc}}}{k_{\text{el}}^{n}}\, v_{\text{prod}}(t)^{n}. \qquad \text{(S7)}$$

Integrating over the main window yields

$$N(t_*)^{n+1} - N(t_\mathrm{w})^{n+1} \propto \int_{t_\mathrm{w}}^{t_*} v_\mathrm{prod}(t)^n \, dt. \qquad \text{(S8)}$$

The term $N(t_\mathrm{w})^{n+1}$ is an initial-condition contribution at the onset of the window. In practice it can be negligible relative to $N(t_*)^{n+1}$ or absorbed into the prefactor without affecting the scaling exponents; thus, we write the dominant scaling as

$$N(t_*)^{n+1} \propto \int_{t_\mathrm{w}}^{t_*} v_\mathrm{prod}(t)^n \, dt. \qquad \text{(S8')}$$

Using $[\mathrm{Thr}]_\mathrm{eff}(t) = [\mathrm{Thr}]_0 \, a(t)$ in Eq. (S1),

$$v_\mathrm{prod}(t)^n = (k_\mathrm{cat}[\mathrm{Thr}]_0)^n \, a(t)^n \left(\frac{F(t)}{K_m + F(t)}\right)^n. \qquad \text{(S9)}$$

Therefore,

$$N(t_*)^{n+1} \propto [\mathrm{Thr}]_0^n \, I(F_0; K_m, t_\mathrm{w}, t_*), \qquad \text{(S10)}$$

where the window functional is

$$I(F_0; K_m, t_\mathrm{w}, t_*) \equiv \int_{t_\mathrm{w}}^{t_*} a(t)^n \left(\frac{F(t)}{K_m + F(t)}\right)^n dt. \qquad \text{(S11)}$$

S1.5. Relation between K_m and our concentration range; linear-Michaelis-Menten regime during the main window

In our experiments, the initial fibrinogen concentrations are 0.36–1.45 mg/mL for purified and 0.36–2.9 mg/mL for plasma. Using M_W(Fgn) $\approx$ 340 kDa, these correspond to

$$0.36, 0.73, 1.45, 2.9\, mg/mL \;\Rightarrow 1.06, 2.15, 4.26, 8.53\, \mu M. \qquad \text{(S12)}$$

Using previous reported K_m (a few μM) [5], the initial substrate level F_0 can be comparable to K_m at $t = 0$. However, because substrate is depleted (Eq. S2), and because the dominant structural contribution is assumed to arise after an initial transient once the system has entered the main window $[t_\mathrm{w}, t_*]$ (Eq. S6), so that the substrate level sampled during this window lies in the low-substrate regime:

$$F(t) \ll K_m, t \in [t_\mathrm{w}, t_*], \qquad \text{(S13)}$$

then the Michaelis–Menten factor is well approximated by its linear form

$$\frac{F(t)}{K_m + F(t)} \approx \frac{F(t)}{K_m}. \qquad \text{(S14)}$$

Under this linearization, the depletion dynamics are effectively linear in F over the window, so different initial conditions approximately rescale the substrate time course:

$$F(t) = F_0\, g(t), t \in [t_{\mathrm{w}}, t_*], \qquad \text{(S15)}$$

where $g(t)$ represents a shared temporal shape (set by $[\mathrm{Thr}]_0$ and $a(t)$) that is approximately independent of F_0 over the finite range studied.

Substituting Eqs. (S14)–(S15) into Eq. (S11) yields

$$I(F_0; K_m, t_{\mathrm{w}}, t_*) \approx \left(\frac{F_0}{K_m}\right)^n \int_{t_{\mathrm{w}}}^{t_*} a(t)^n\, g(t)^n\, dt \equiv \left(\frac{F_0}{K_m}\right)^n J([\mathrm{Thr}]_0; t_{\mathrm{w}}, t_*), \qquad \text{(S16)}$$

with

$$J([\mathrm{Thr}]_0; t_{\mathrm{w}}, t_*) \equiv \int_{t_{\mathrm{w}}}^{t_*} a(t)^n g(t)^n dt. \qquad \text{(S17)}$$

Combining Eqs. (S10) and (S16) gives the scaling

$$N(t_*) \propto [\mathrm{Thr}]_0^{\frac{n}{n+1}}\, F_0^{\frac{n}{n+1}}. \qquad \text{(S18)}$$

S1.6. Geometric allocation and emergence of the final power law (example: diameter)

At readout time t_* in a fixed observation volume, the total incorporated fibrin mass scales to leading order with the initial fibrinogen supply:

$$M \propto F_0. \qquad \text{(S19)}$$

Assuming uniform density and equal mass allocation across $N(t_*)$ growth units,

$$M \propto N(t_*)\, d^2\, \ell(t_*), \qquad \text{(S20)}$$

where d is fiber diameter and $\ell(t_*)$ is an effective length contributed per growth unit at readout. If ℓ varies weakly across the matrix or follows an effective power law

$$\ell(t_*) \propto F_0^u\, [\mathrm{Thr}]_0^v, \qquad \text{(S21)}$$

then combining Eqs. (S18)–(S21) yields the multiplicative power law

$$d \propto F_0^\alpha\, [\mathrm{Thr}]_0^\beta, \qquad \text{(S22)}$$

with

$$\alpha = \frac{1}{2}\left(1 - \frac{n}{n+1} - u\right) = \frac{1}{2}\left(\frac{1}{n+1} - u\right), \qquad \text{(S23)}$$

$$\beta = -\frac{1}{2}\left(\frac{n}{n+1} + v\right). \qquad \text{(S24)}$$

S2. Structural Median or Mean Data for each Fibrinogen and Thrombin Concentration

S2.1. Fiber Diameter

Fgn (mg/mL)	Thr (U/mL)	Plasma Fiber Diameter Median [IQR] (n=16)	Purified Fiber Diameter Median [IQR] (n=16)
0.36	0.05	130 [127, 136]	100 [98, 107]
0.36	0.1	128 [114, 138]	91 [89, 93]
0.36	0.2	100 [92, 113]	76 [73, 78]
0.36	1	61 [59, 67]	
0.73	0.05	142 [140, 151]	116 [110, 119]
0.73	0.1	131 [126, 138]	102 [99, 108]
0.73	0.2	117 [111, 127]	83 [81, 87]
0.73	1	72 [69, 77]	
1.45	0.05	160 [150, 171]	125 [117, 127]
1.45	0.1	159 [147, 167]	115 [107, 121]
1.45	0.2	130 [125, 136]	93 [92, 96]
1.45	1	91 [87, 93]	
2.9	0.05	190 [177, 195]	
2.9	0.1	162 [144, 186]	
2.9	0.2	154 [145, 168]	
2.9	1	98 [93, 115]	

Table S1: Median fiber diameters for plasma and purified fibrin networks, calculated from per-image medians across 16 analyzed images; interquartile ranges (IQR) are reported for each condition.

S2.2. Fiber Length

Fgn (mg/mL)	Thr (U/mL)	Plasma Fiber Length Median [IQR] (n=16)	Purified Fiber Length Median [IQR] (n=16)
0.36	0.05	2143 [1942, 2414]	1686 [1608, 1811]
0.36	0.1	1750 [1567, 1940]	1328 [1210, 1483]
0.36	0.2	1624 [1455, 1969]	1114 [974, 1216]
0.36	1	1093 [1016, 1216]	
0.73	0.05	2302 [1836, 2726]	1786 [1617, 1910]
0.73	0.1	1848 [1694, 2189]	1507 [1285, 1605]
0.73	0.2	1554 [1430, 1726]	1262 [1123, 1367]
0.73	1	1087 [995, 1251]	
1.45	0.05	2379 [2267, 2664]	1979 [1730, 2029]
1.45	0.1	1934 [1713, 2267]	1516 [1399, 1594]
1.45	0.2	1745 [1571, 1876]	1259 [1135, 1329]
1.45	1	1162 [1019, 1299]	
2.9	0.05	2528 [2173, 2781]	
2.9	0.1	2119 [2076, 2373]	
2.9	0.2	1891 [1762, 1968]	
2.9	1	1297 [1170, 1384]	

Table S2: Median fiber lengths for plasma and purified fibrin networks, calculated from per-image medians across 16 analyzed images; interquartile ranges (IQR) are reported for each condition.

S2.3. Fiber Density

Fgn (mg/mL)	Thr (U/mL)	Plasma Fiber Density Mean ± SD (n=36)	Purified Fiber Density Mean ±SD (n=36)
0.36	0.05	5.1 ± 0.8	7.3 ± 0.8
0.36	0.1	5.9 ± 0.9	10.0 ± 1.4
0.36	0.2	9.7 ± 1.2	15.1 ± 1.4
0.36	1	21 ± 2	
0.73	0.05	10.2 ± 2.1	12.7 ± 1.2
0.73	0.1	10.7 ± 0.9	16.0 ± 1.5
0.73	0.2	11.9 ± 1.2	22 ± 2
0.73	1	24 ± 2	
1.45	0.05	14 ± 2	22 ± 2
1.45	0.1	15.5 ± 1.6	29 ± 2
1.45	0.2	14.0 ± 1.6	36 ± 2
1.45	1	27 ± 3	
2.9	0.05	18 ± 2	
2.9	0.1	20 ± 5	
2.9	0.2	22 ± 4	
2.9	1	32 ± 4	

Table S3: Mean fiber densities for plasma and purified fibrin networks, calculated from per-image means across 36 analyzed images; standard deviation (SD) are reported for each condition.

S2.4. Bubble Diameter

Fgn (mg/mL)	Thr (U/mL)	Plasma Bubble Diameter Median [IQR] (n=36)	Purified Bubble Diameter Median [IQR] (n=36)
0.36	0.05	13.7 [12.9, 13.9]	12.0 [11.4, 12.5]
0.36	0.1	13.1 [11.4, 13.6]	11.1 [10.8, 11.5]
0.36	0.2	10.5 [10.2, 11.2]	8.9 [8.6, 9.3]
0.36	1	7.0 [6.8, 7.2]	
0.73	0.05	10.1 [10.0, 10.3]	8.5 [8.2, 8.7]
0.73	0.1	9.9 [9.6, 10.3]	8.2 [7.7, 8.4]
0.73	0.2	9.3 [8.6, 9.7]	7.6 [7.3, 7.8]
0.73	1	6.7 [6.6, 6.9]	
1.45	0.05	8.6 [8.0, 9.0]	6.6 [6.4, 6.8]
1.45	0.1	7.7 [7.0, 8.0]	5.9 [5.8, 6.0]
1.45	0.2	6.9 [6.3, 7.0]	5.60 [5.56, 5.64]
1.45	1	6.4 [6.2, 6.5]	
2.9	0.05	7.2 [6.6, 8.1]	
2.9	0.1	6.9 [6.5, 7.2]	
2.9	0.2	6.6 [6.4, 6.9]	
2.9	1	5.9 [5.7, 6.1]	

Table S4: Median bubble diameter for plasma and purified fibrin networks, calculated from per-image medians across 36 analyzed images; interquartile ranges (IQR) are reported for each condition.

S3. Additional Analysis to Test Robustness of Statistics (Leave-One-Condition-Out Analysis)

**Plasma**		**Value**	**Leave-One-Condition-Out**
**Parameters for Fiber diameter**	k	81	[79, 84]
	α	0.19	[0.17, 0.20]
	β	- 0.23	[-0.25, -0.21]
**Parameters for Fiber length**	k	1155	[1144, 1166]
	α	0.09	[0.08, 0.09]
	β	-0.24	[-0.24, -0.23]
**Parameters for Fiber density**	k	25	[22, 25]
	α	0.45	[0.40, 0.48]
	β	0.30	[0.26, 0.32]
**Parameters for Bubble diameter**	k	6.6	[6.5, 6.6]
	α	-0.24	[-0.25, -0.22]
	β	-0.14	[-0.15, -0.13]

Table S5: Power-law fitting parameters and leave-one-condition-out ranges for plasma clots

**Purified Fibrinogen**		**Value**	**Leave-One-Condition-Out**
**Parameters for Fiber diameter**	k	64	[61, 65]
	α	0.15	[0.15, 0.17]
	β	- 0.21	[-0.23, -0.21]
**Parameters for Fiber length**	k	775	[741, 797]
	α	0.07	[0.07, 0.11]
	β	-0.29	[-0.31, -0.28]
**Parameters for Fiber density**	k	57	[51, 59]
	α	0.73	[0.69, 0.75]
	β	0.43	[0.38, 0.44]
**Parameters for Bubble diameter**	k	5.1	[4.9, 5.5]
	α	-0.41	[-0.44, -0.39]
	β	-0.14	[-0.16, -0.11]

Table S6: Power-law fitting parameters and leave-one-condition-out ranges for purified fibrinogen clots

S4. Turbidity Analysis of Fibrin Networks Formation with Different Amounts of Added Alexa-488-Labeled Fibrinogen

Methods. Turbidity-based clot formation assays were performed using a SpectraMax microplate reader by monitoring absorbance at 405 nm every 7 s for 3 h in 96-well microplates (Corning flat clear-bottom white polystyrene, TC-treated). Plasma clots were prepared using fibrinogen concentrations of 0.73, 1.45, or 2.4 mg/mL with thrombin fixed at 0.2 U/mL. To evaluate the effect of fluorescent labeling on clot formation, Alexa Fluor 488-labeled fibrinogen was incorporated at 0%, 1.5%, 3%, 6%, or 12% of the total fibrinogen concentration.

Each curve is the average of two separate experiments.

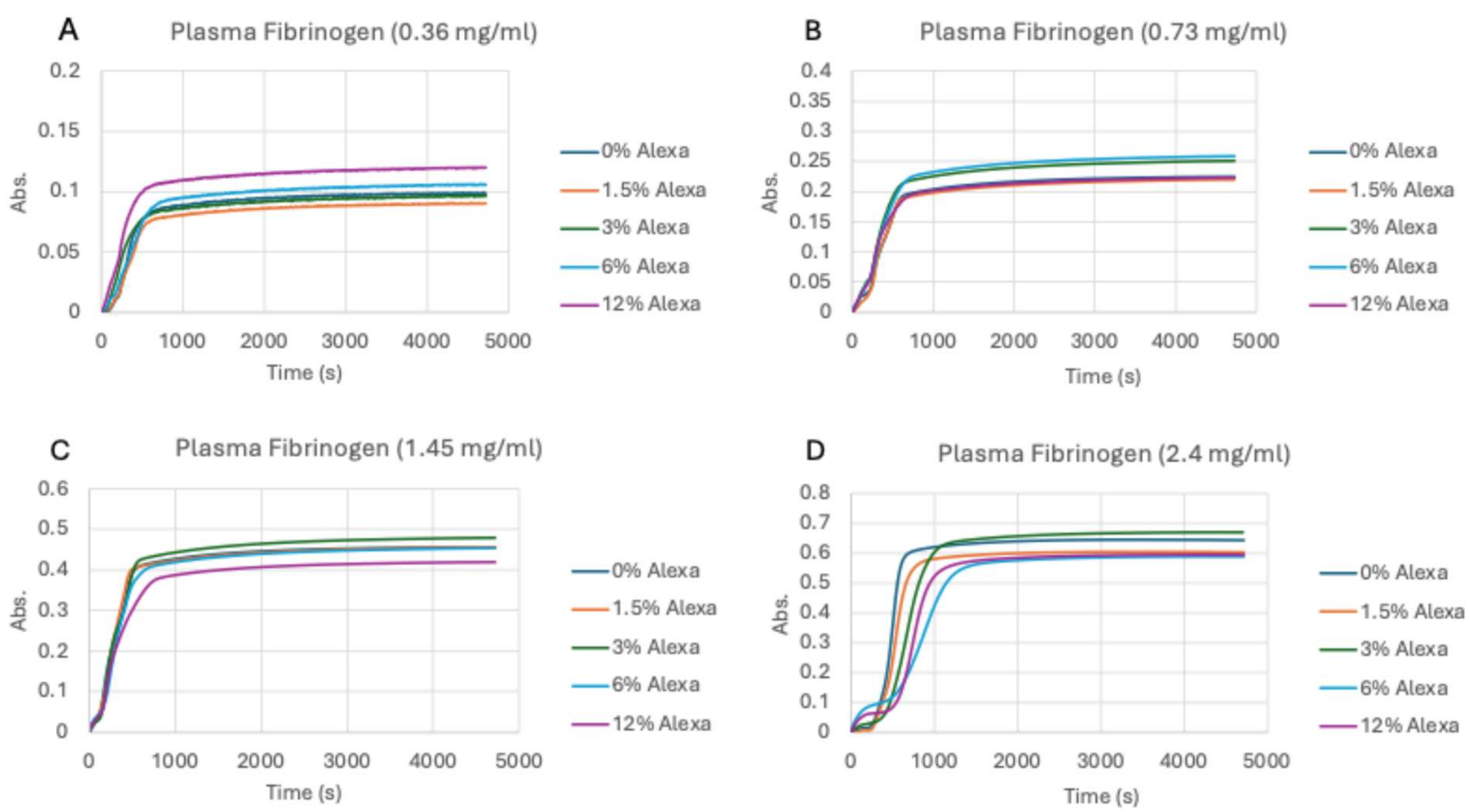

Figure S1. Turbidity curves for plasma fibrinogen concentrations ranging from 0.36 to 2.4 mg/mL with 0–12% Alexa Fluor 488 labeled fibrinogen added.

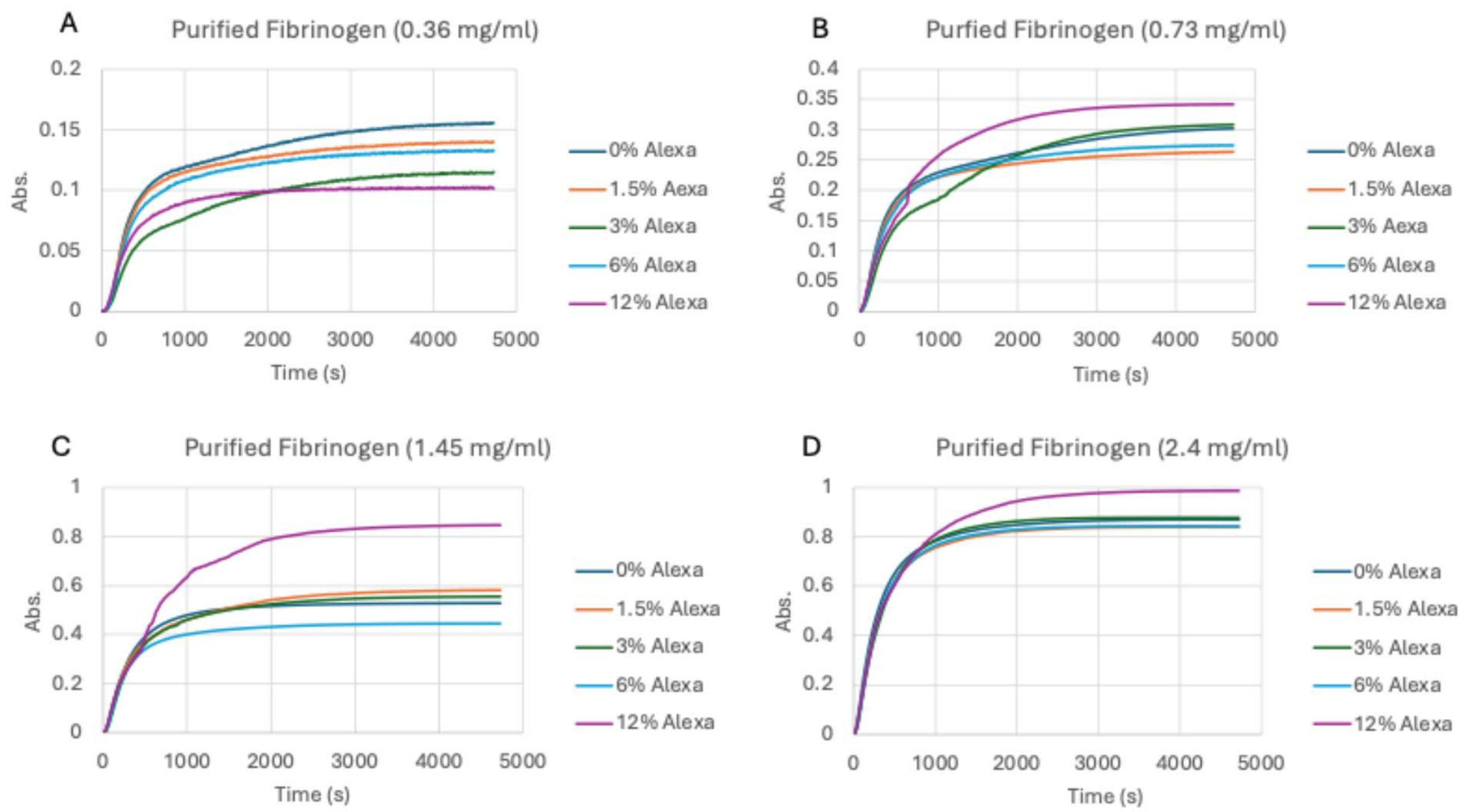

Figure S2. Turbidity curves for purified fibrinogen concentrations ranging from 0.36 to 2.4 mg/mL with 0–12% Alexa Fluor 488 labeled fibrinogen added.

Minimal differences in fibrin formation dynamics and final turbidity were observed up to 6% labeling, whereas more noticeable deviations appeared at 12% labeling.

References

[1] J. Jesty, The Kinetics of Inhibition of Thrombin by Antithrombin in the Presence of Components of the Hemostatic System, Blood 66(5) (1985) 1189-1195.

[2] J.W. Weisel, C. Nagaswami, Computer Modeling of Fibrin Polymerization Kinetics Correlated with Electron-Microscope and Turbidity Observations - Clot Structure and Assembly Are Kinetically Controlled, Biophysical Journal 63(1) (1992) 111-128.

[3] A.L. Fogelson, J.P. Keener, Toward an understanding of fibrin branching structure, Phys Rev E 81(5) (2010) 051922, 1-9.

[4] L.A. Segel, M. Slemrod, The Quasi-Steady-State Assumption - a Case-Study in Perturbation, Siam Rev 31(3) (1989) 446-477.

[5] D.L. Higgins, S.D. Lewis, J.A. Shafer, Steady-State Kinetic-Parameters for the Thrombin-Catalyzed Conversion of Human-Fibrinogen to Fibrin, J Biol Chem 258(15) (1983) 9276-9282.
